# Hypofractionated Radiotherapy and Stereotactic Boost with Concurrent and Adjuvant Temozolamide for Glioblastoma in Good Performance Status Elderly Patients – Early Results of a Phase II Trial

**DOI:** 10.3389/fonc.2012.00122

**Published:** 2012-10-16

**Authors:** Scott R. Floyd, Ekkehard M. Kasper, Erik J. Uhlmann, Ekokobe Fonkem, Eric T. Wong, Anand Mahadevan

**Affiliations:** ^1^Beth Israel Deaconess Medical CenterBoston, MA, USA; ^2^Harvard Medical SchoolBoston, MA, USA

**Keywords:** glioblastoma, stereotactic radiation, temozolamide

## Abstract

Glioblastoma Multiforme (GBM) is an aggressive primary brain neoplasm with dismal prognosis. Based on successful phase III trials, 60 Gy involved-field radiotherapy in 30 fractions over 6 weeks [Standard radiation therapy (RT)] with concurrent and adjuvant temozolomide is currently the standard of care. In this disease, age and Karnofsky Performance Status (KPS) are the most important prognostic factors. For elderly patients, clinical trials comparing standard RT with radiotherapy abbreviated to 40 Gy in 15 fractions over 3 weeks demonstrated similar outcomes, indicating shortened radiotherapy may be an appropriate option for elderly patients. However, these trials did not include temozolomide chemotherapy, and included patients with poor KPS, possibly obscuring benefits of more aggressive treatment for some elderly patients. We conducted a prospective Phase II trial to examine the efficacy of a hypofractionated radiation course followed by a stereotactic boost with concurrent and adjuvant temozolomide chemotherapy in elderly patients with good performance status. In this study, patients 65 years and older with a KPS > 70 and histologically confirmed GBM received 40 Gy in 15 fractions with 3D conformal technique followed by a 1–3 fraction stereotactic boost to the enhancing tumor. All patients also received concurrent and adjuvant temozolomide. Patients were evaluated 1 month post-treatment and every 2 months thereafter. Between 2007 and 2010, 20 patients (9 males and 11 females) were enrolled in this study. The median age was 75.4 years (range 65–87 years). At a median follow-up of 11 months (range 7–32 months), 12 patients progressed and 5 are alive. The median progression free survival was 11 months and the median overall survival was 13 months. There was no additional toxicity. These results indicate that elderly patients with good KPS can achieve outcomes comparable to the current standard of care using an abbreviated radiotherapy course, radiosurgery boost, and temozolomide.

## Introduction

Malignant gliomas, including glioblastoma multiforme (GBM) are the most common primary brain tumors in adults and the age-adjusted incidence of these high-grade gliomas has increased over recent years (Lowry et al., 1998; Kohler et al., 2011). Currently available data extrapolated from retrospective studies or meta-analysis suggest that performance status is the strongest prognostic factor in the elderly (Curran et al., 1993; Li et al., 2011). However, many of these retrospective studies suffer from biased patient selection and often do not include patients over 65 years of age.

Older patients have a worse survival outcome compared with younger patients (Ampil et al., 1992; Siker et al., 2011). Recursive partitioning analysis of Radiation Therapy Oncology Group (RTOG) glioma trials (Curran et al., 1993), the United Kingdom Molecular Research Council (UK MRC), and European Organization for Research and Treatment of Cancer (EORTC) prognostic groups have consistently shown that elderly patients and those with poor performance do poorly. Shortened treatment time may be advantageous for many elderly patients as it potentially maximizes out-of-hospital time in this disease with limited prognosis. The National Cancer Institute of Canada (NCIC) conducted a multi institutional randomized controlled study comparing 40 Gy in 15 fractions over 3 weeks to the standard treatment with 60 Gy in 30 fractions over 6 weeks (Roa et al., 2004). There was no statistically significant difference between the two arms in survival or quality of life, and abbreviated radiation therapy (RT) patients required less steroid therapy. Findings were similar in the analysis of the UK MRC Glioma study where there was no difference in overall survival outcome in the 57 elderly (>65 years subset) patients who received 45 Gy in 20 fractions (Bleehen and Stenning, 1991). These trials included many elderly patients with poor Karnofsky Performance Status (KPS). While the shorter course may be suitable for the elderly, patients with good performance status may benefit from full doses of radiation. One strategy to deliver higher radiation doses while preserving an overall abbreviated treatment course is through stereotactic radiosurgery (SRS).

In early retrospective series the overall median survival of patients treated with SRS was quite encouraging (Buatti et al., 1995; Gannett et al., 1995; Masciopinto et al., 1995). Based on this, the RTOG opened a prospective randomized trial evaluating up front SRS followed by external beam radiation therapy (EBRT) with BCNU vs. EBRT and BCNU in 1993 (protocol 93-05). Results showed no difference in survival outcomes between the study arms (Souhami et al., 2004). Thus the best current evidence does not support an advantage for adding SRS to standard doses of radiotherapy for GBM. However, the benefit of adding SRS to reduced doses remains an open question.

In the case of elderly patients with GBM, we hypothesized that the advantages of hypo fractionation (shorter course of radiation using higher daily doses) could be supplemented by SRS to deliver total doses of radiation more in line with Standard RT. We also reasoned that this dose of radiation when combined with concurrent and adjuvant Temozolamide could approximate results obtained with Standard RT and temozolomide as demonstrated in Phase III randomized trials (Stupp et al., 2005) while preserving the advantages of a shorter overall treatment course.

## Materials and Methods

This was an institutional review board approved prospective single arm phase II study. Patient selection and eligibility criteria required that all patients have histologically confirmed GBM, be 65 years of age or older, have a pre-treatment KPS of >70, and not have contraindications for radiotherapy or temozolomide. Pathology was confirmed with surgical resection when deemed feasible; otherwise a core needle biopsy was obtained. Pre- and post-operative (when resected) MRI along with baseline blood counts and chemistry studies were obtained in all patients. Patients with recurrent glioma, brainstem invasion, and prior radiation to the head and neck area were excluded from the study. Protocol therapy was started within 5 weeks of surgery.

### External beam radiation therapy

Patients were simulated in a supine position with a thermoplastic immobilization head mask. Planning CT scans with IV contrast were obtained and transferred to the treatment planning station. Pre-operative and post-operative MRI scans were utilized for fusion with simulation CT scans for outlining the target volume. Fused FLAIR/T2 images were used to contour changes in the fused axial CT images to delineate the gross tumor volume (GTV). A 1.5–2 cm expansion was added to the GTV to create the planning target volume (PTV). Non-coplanar egocentric beams with appropriate energy and conformal collimation were used. Isodose distributions, treatment plans, and dose volume histograms were generated to produce a homogenous plan with less than ±10% inhomogeneity within the target volume. The prescribed dose to the PTV was 40 Gy delivered in 15 daily fractions.

### Stereotactic radiosurgery

Frameless SRS with the CyberKnife system was used to deliver the boost. The target volume was determined by changes in the contrast enhancing T1 MRI. The isodose line covering 95% of the target volume was used as the prescription. The prescribed dose depended on the greatest dimension of the lesion. For lesions less then 2 cm the dose was 22 Gy, for lesions between 2.1 and 3.0 cm the dose was 18 Gy, for lesions between 3.1 and 4.1 cm the dose was 15 Gy, and for lesions larger than 4.1 a fractionated regime of 8 Gy × 3 fractions for a total dose of 24 Gy was used. All patients received anti-edema (dexamethasone) and anti-seizure (leveteracetam) prophylaxis.

### Dose limitation for critical structures

The lens and cervical spine were shielded from the direct beam during EBRT delivery. When possible, without shielding the gross tumor, attempts were made to limit the optic chiasm dose to 54 Gy, the retina dose (of at least one, but preferably both eyes) to 50 Gy, the lens dose to 8 Gy, and the brain stem dose to 60 Gy in 2 Gy/fraction equivalents and inclusive of the radiosurgery boost dose.

### Temozolomide chemotherapy

Temozolomide was prescribed orally (75 mg/m^2^/day) for 4 weeks during EBRT and during the week of SRS boost therapy. Chemotherapy began 1 h before the first fraction of EBRT and continued during weekends and holidays. Four to six weeks after completion of SRS, Temozolomide (150 mg/m2/day) was prescribed orally for 5 days (days 1–5) of each 28-day cycle. This schedule was continued without interruption, as long as there was no tumor progression and toxicity was ≤grade 3, for 1 year or until completion of 28 treatment cycles (whichever was longer).

### Follow-up

All patients were followed with weekly clinical examination and complete blood counts during treatment. Patients were then routinely followed every month for blood counts, clinical assessment, and Temozolamide prescription. Contrast enhanced MRI scans were obtained 1 month after radiation and every 2 months thereafter until progression. Progression was defined as worsening enhancement in MRI in the setting of neurological deterioration.

### Statistical analysis

The primary end point of the study is median and 6 month progression free survival with a secondary end point of overall survival and tolerability. The study was designed to discern a 50% improvement in progression free survival at 6 months over that reported in Roa et al. (2004). Calculations indicated that a sample size of 25 patients were required for a single arm phase II study. Descriptive statistics were used to describe the data. Kaplan–Meier survival analysis was used for computing progression free and overall survival with Prism (GraphPad Software Inc., La Jolla, CA, USA).

## Results

**Figure 1:**
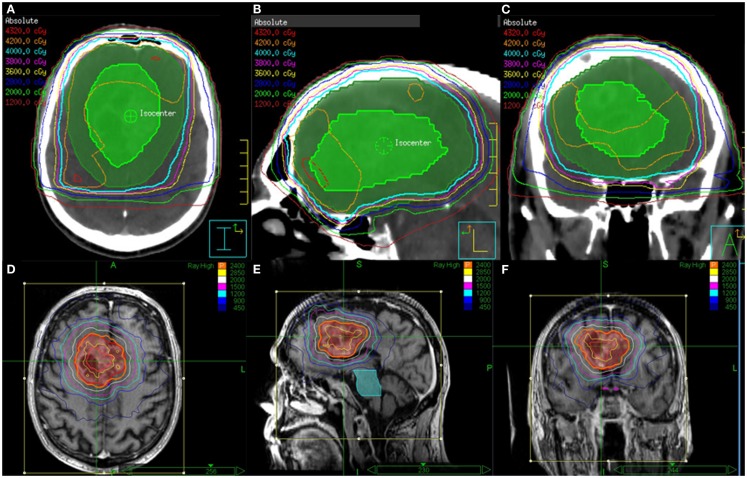
**EBRT and CyberKnife SRS treatment plans for a patient who received 40 Gy in 15 fractions to FLAIR for the first course followed an SRS boost to T1 Enhancement at a total dose of 24 Gy delivered in 3 fractions**. Shown are the **(A)** axial, **(B)** sagittal, and **(C)** coronal views of the EBRT treatment plans and the **(D)** axial, **(E)** sagittal, and **(F)** coronal views of the CyberKnife SRS treatment plans.

Twenty patients (9 male and 11 female patients) were included in this study. The mean age was 75.4 years (range 65–87 years). All patients completed the protocol treatment as prescribed. After delivery of 40 Gy in 15 fractions for the initial course, three patients received a single fraction SRS boost. The remaining 17 patients had a target volume exceeding 4 cm in greatest dimension. These patients received a total SRS boost dose of 24 Gy delivered in 3 fractions. Table 1 describes the patient and treatment characteristics. Figure 1 illustrates an EBRT and a SRS treatment plan.

**Table 1:** **Patient and treatment characteristics**.

Sex
Male	9
Female	11
Age (>65 years) – mean (range)	75.4 years (65–87 years)
Extent of surgery
Gross total resection	10
Sub total resection	3
Biopsy	7
Karnofsky performance status
70	14
80	2
90	4
100	0
Salvage therapy
Bevacizumab	5
Phase I trial	2
Other systemic therapy	0
Stereotactic radiosurgery	1
No salvage therapy	9
N/A (no progression)	3

**Figure 2:**
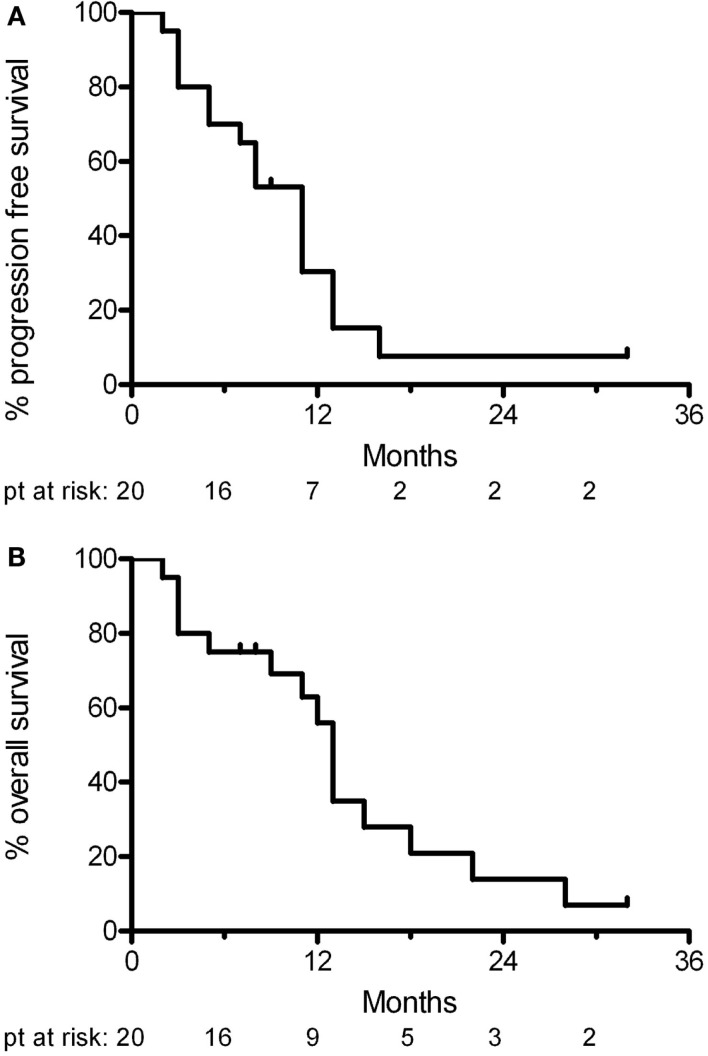
**Kaplan–Meier analysis of (A) progression free survival and (B) overall survival**.

At a median follow-up of 11 months (range 7–32 months), 11 patients had tumor progression. Four of these patients deteriorated neurologically in the absence radiological progression. Two patients died of unrelated causes (one from untreated sepsis and the other from a pulmonary embolism from deep venous thrombosis). Five patients were still alive at last follow-up. The median progression free survival was 11 months and the median overall survival was 13 months (Figure 2). At progression, patients were offered second line chemotherapy or hospice care.

### Toxicity

All patients tolerated and completed protocol treatment. All patients experienced fatigue and skin reaction (erythema and alopecia), not requiring and further treatment (Grade I). Four patients required prolonged dexamethasone for symptomatic cerebral edema, which eventually resolved (20% grade II toxicity) in all but two of the patents. These two patients required hospitalization for management. Two additional patients were hospitalized: one for urinary tract infection, and the other for pulmonary embolism. These events were unlikely to be directly related to study participation. Overall four patients (20%) experienced Grade III toxicity. There were no life threatening (Grade IV) complications or treatment related deaths.

## Discussion

The current standard treatment for patients with glioblastoma is 60 Gy of involved-field radiation in 30 fractions delivered over 6 weeks with concurrent and adjuvant Temozolamide. The 6 week duration of this conventional radiation treatment is not well tolerated by the elderly. While lesser doses of radiation have been shown to be equivalent in the elderly with a wide range in performance status, for those patients with a good performance status (KPS > 70) it is possible that conventional radiation doses may be more efficacious. Hence hypofractionation (shorter course of radiation with larger doses per fraction) along with a highly conformal boost could potentially deliver equivalent, but tolerable doses of radiation in short treatment duration with improved outcome and quality of life. To date, this study supports that hypothesis with a median progression free survival of 11 months and a median overall survival of 13 months.

The elderly population is growing and with it the incidence of cancer, especially brain tumors, is increasing in the elderly age group. However, it is unclear whether this increase is a result of improvements in diagnosis or represents a true increase in the incidence of cancer (Lowry et al., 1998; Kohler et al., 2011). Older patients have a worse survival outcome compared with younger patients. Several hypotheses have been put forward to explain the poor clinical outcome of elderly patients with brain tumors. The presence of co-morbidity, resistance to cancer therapy, genetic aberrations, different histology, neurodegeneration, and age discrimination have all been proposed as reasons why elderly patients have a poorer outcome compared with younger patients. However, this does not explain why survival is also reduced in elderly patients without co-morbidities. Brain tumors in elderly patients seem to have an intrinsic resistance to treatment. Rosenblum et al. (1982) observed that sensitivity to carmustine in clonogenic cells from biopsy specimens was strongly correlated with patient age. Elderly patients may also develop larger tumors as a result of cerebral atrophy, which can allow more tumor growth before symptoms become evident.

In a study of post-operative RT for 301 newly diagnosed GBM patients, Barker et al. (2001) reported that younger age (*P* < 0.006), higher post-operative KPS before RT (*P* < 0.027), and more extensive resection (*P* < 0.028) were strictly correlated with response to RT, which is known to be a strong predictor of survival. In one prospective non-randomized study of 79 GBM patients over 65 years of age who had minimal residual disease, good performance status (KPS > 60), and received standard of care treatment, overall median survival was 12.5 months (comparable to other reports in similar groups of patients; Brandes and Monfardini, 2003; Brandes et al., 2003). This study showed that for elderly patients, aggressive management with surgical resection followed by RT (59.4 Gy/33 fractions with limited fields) and adjuvant temozolomide provided a significant survival advantage over RT alone (median, 14.9 vs. 11.2 months; *P* < 0.002). The randomized NCIC and the MRC trials discussed above favored a shortened course of radiation for elderly patients, however, the median overall survival in this trial was diminished when compared to standard of care therapy at 5.1 months for Standard RT and 5.6 months for abbreviated course RT. The two caveats in these trials were that many patients likely had a poor KPS and the patients did not receive systemic therapy. Hence it is possible that in the chemotherapy era those patients with good KPS may benefit from full doses of radiation.

The 1990s saw published reports on several small series of patients treated with SRS boost for the primary treatment of malignant glioma (Buatti et al., 1995; Gannett et al., 1995; Masciopinto et al., 1995; Kondziolka et al., 1997; Shenouda et al., 1997; Shrieve et al., 1999; Nwokedi et al., 2002). However, the results of radiosurgical series were viewed with skepticism (Anker et al., 2010). In an attempt to reduce bias, many authors sought out retrospective control populations. One of the largest reviews attempted to retrospectively stratify 115 patients from three institutions according to the prognostic classes of the RTOG recursive partitioning analysis of the patients enrolled on RTOG 74-01, 79-18, and 82-02 (Sarkaria et al., 1995). This analysis concluded that there was a significant improvement in both 2 year and median survival favoring SRS-treated patients. Nevertheless, this approach, along with all retrospective comparisons, is inherently flawed. RTOG opened protocol 93-05 in 1993 (Souhami et al., 2004). This was a prospective randomized trial evaluating upfront SRS followed by EBRT with BCNU (Arm 1) vs. EBRT and BCNU (Arm 2). At a median follow-up of 44 months, the median survival for Arm 1 was 14.1 months (95% CI: 11.0–14.9) and 13.7 months (95% CI: 11.3–15.2) for Arm 2 (*P* = 0.53). The 2-year actuarial survival rates were 22% for Arm 1 and 16% for Arm 2. There was no statistically significant difference in the incidence of late toxicity between the arms. Hence, the current standard therapy remains 6 weeks of radiation (60 Gy) with concurrent and adjuvant temozolamide (Stupp et al., 2005).

Our trial aimed at achieving the benefit of delivering adequate radiation in a shortened time with concurrent and adjuvant chemotherapy. The progression free survival in this trial of 11 months compares very favorably to the EORTC/NCIC trial and to that of prior trials for this disease. This is in spite of our trial’s designed inclusion of only elderly patients rather than patients of all ages. This evidence hints that our abbreviated overall course of treatment is not inferior to standard of care treatment.

The steroid dependency rate in out trial was about 20%. In two French studies (Marantidou et al., 2010; Carpentier et al., 2012) with conventional radiation, about 55% of patients required increasing doses of steroids and 40–100% of patients had persistent requirement of steroids at 3 months depending on extent of resection. Also in our study steroids were deliberately used as prophylaxis for the stereotactic boost. There was no other excessive toxicity in our study other than Grade II fatigue, skin erythema, and alopecia (Table 2).

**Table 2:** **Toxicity**.

Toxicity	Number (%)	Grade
Skin erythema	21(100)	I
Patchy alopecia	21(100)	II
Steroid dependence	2(9.5)	II
Cerebral edema (hospitalization)	2(9.5)	III
Possibly related (UTI, PE)	2(9.5)	III

*UTI, urinary tract infection; PE, pulmonary embolism*.

One theoretical reason for the improved local control, as reflected in improved progression free survival, could be the delivery of higher stereotactic radiation dose to the contrast enhancement after initial radiation to the surrounding edema. It has been hypothesized that treatment resistant glioma stem cells reside in the areas of enhancement and these may be better treated with higher doses of radiation in the setting of systemic therapy (Cheng et al., 2010). This hypothesis remains to be proven.

## Conclusion

Old age and poor performance status are the two most significant prognostic factors in patients with Glioblastoma. Elderly patients tolerate a prolonged course of radiation poorly and those with good performance status may benefit from aggressive therapy. Hypofractionated radiation with a stereotactic boost can deliver a shorter course of adequate radiotherapy effectively and safely in the temozolamide era.

## Conflict of Interest Statement

The authors declare that the research was conducted in the absence of any commercial or financial relationships that could be construed as a potential conflict of interest.

## Figures and Tables

**Figure A1:**
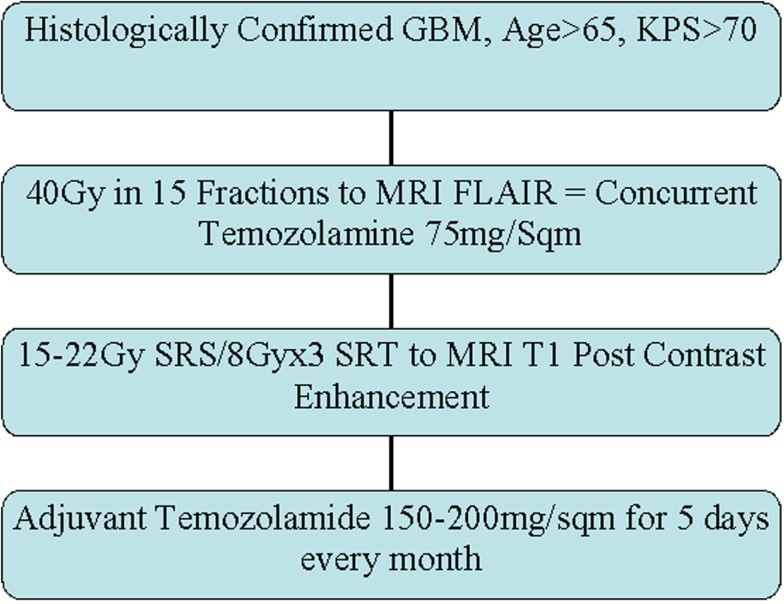
**Trial profile**.
